# Supplementary material for: Continuous millisecond conformational cycle of a DEAH box helicase reveals control of domain motions by atomic-scale transitions
Source: Commun Biol. 2023 Apr 7;6:379. doi: 10.1038/s42003-023-04751-z (PMC10082070; doi:10.1038/s42003-023-04751-z)
Supplement: Supplementary file 2 — Supplementary Material [file 42003_2023_4751_MOESM2_ESM.pdf]

Supplementary Information: Continuous millisecond conformational cycle of a DEAH box helicase reveals control of domain motions by atomic-scale transitions

Robert A. Becker [1], Jochen S. Hub [1]

[1] *Theoretical Physics and Center for Biophysics, Universität des Saarlandes, Germany*

Supplementary Table S1. Key properties of the adaptive sampling rounds.

For each round of adaptive sampling, sum of simulation times t_{sim}, number of simulations N_{sim}, number of observed transitions N_{trans}, estimated transition rate k for the opening (left) and closing process (right).

	Opening				Closing			
AS round	t_{sim} (µs)	N_{sim}	N_{trans}	k (1/µs)	t_{sim} (µs)	$\mathrm{N_{sim}}$	N_{trans}	k (1/µs)
1	10.0	100	1	0.10	0.8	10	3	3.69
2	10.0	100	1	0.10	0.7	10	0	–
3	10.0	100	9	0.90	0.7	10	0	–
4	10.0	100	0	–	2.0	30	1	0.29
5	5.0	500	2	0.13	3.0	30	0	–
6	8.3	100	20	2.41	2.0	30	2	0.40
7	0.4	40	0	–	2.0	20	2	1.00
8	0.3	40	0	–	4.0	40	0	–
9	1.6	40	1	0.43	5.0	50	5	0.56
10	–	–	–	–	5.0	50	0	–
11	–	–	–	–	4.0	40	0	–
12	–	–	–	–	3.5	40	0	–
13	–	–	–	–	2.7	40	1	0.06

	RecA-Dist.	G349-U5	T381-U5	R435-ATP	K403-U4	E316-U4	S387-Mg	S387-U5	G349-U4-O1	G349-U4-O2	T381-U4-O1	T381-U4-O2	R180-H	R153-H	R180-C-U7	K403-U3	S387-Phi	S387-Psi
E-value	2.65	0.7	0.81	0.38	1.0	0.7	0.26	1.58	0.17	0.32	0.16	0.4	0.18	0.19	0.9	0.33	59.0	-47.0
S-value	3.1	0.19	0.26	0.75	0.29	0.21	1.44	0.2	0.92	0.73	0.9	0.73	0.83	0.62	0.48	0.87	-70.0	-40.0
run1	2.7	0.63	0.75	0.4	1.01	0.82	0.52	1.23	0.18	0.33	0.17	0.41	0.23	0.18	1.04	0.27	-131.14	149.37
run2	2.74	0.64	0.81	0.39	0.9	0.8	0.5	1.21	0.19	0.31	0.18	0.42	0.2	0.19	1.05	0.28	-161.8	154.18
run3	2.71	0.6	0.8	0.38	0.96	0.78	0.56	1.11	0.18	0.33	0.18	0.41	0.2	0.19	1.06	0.27	-151.21	150.41
run4	2.73	0.62	0.78	0.39	0.89	0.82	0.49	1.22	0.18	0.31	0.18	0.43	0.2	0.18	1.05	0.27	-160.6	153.45
run5	2.75	0.62	0.82	0.4	1.03	0.78	0.5	1.16	0.18	0.32	0.17	0.42	0.2	0.19	1.05	0.28	-148.62	152.54
run6	2.77	0.63	0.75	0.4	0.95	0.83	0.56	1.23	0.19	0.31	0.18	0.41	0.21	0.18	1.03	0.27	-162.34	147.8
run7	2.76	0.61	0.77	0.39	0.96	0.8	0.52	1.14	0.18	0.32	0.17	0.42	0.2	0.19	1.06	0.27	-148.18	150.75
run8	2.72	0.63	0.79	0.39	0.9	0.82	0.51	1.23	0.19	0.31	0.18	0.42	0.22	0.19	1.04	0.28	-161.9	150.54
run9	2.74	0.62	0.78	0.4	0.94	0.8	0.5	1.23	0.18	0.32	0.18	0.42	0.21	0.18	1.02	0.27	-147.2	152.26
run10	2.73	0.63	0.8	0.4	0.9	0.83	0.52	1.25	0.19	0.3	0.18	0.43	0.21	0.18	1.0	0.28	-163.25	150.81
run11	2.74	0.6	0.8	0.39	0.93	0.8	0.53	1.12	0.18	0.31	0.18	0.42	0.2	0.19	1.03	0.27	-164.92	150.29
run12	2.73	0.6	0.79	0.38	0.96	0.83	0.54	1.13	0.18	0.32	0.18	0.42	0.21	0.18	1.06	0.28	-130.48	138.06
run13	2.72	0.64	0.78	0.4	0.94	0.77	0.51	1.23	0.18	0.33	0.18	0.42	0.22	0.18	1.05	0.28	-160.61	148.87
run14	2.76	0.64	0.8	0.39	0.92	0.81	0.56	1.25	0.19	0.32	0.17	0.42	0.2	0.18	0.99	0.27	-145.06	151.91
run15	2.72	0.61	0.78	0.4	0.94	0.81	0.53	1.24	0.18	0.32	0.17	0.41	0.2	0.18	1.06	0.28	-112.83	149.76
run16	2.72	0.63	0.84	0.38	0.95	0.81	0.55	1.12	0.19	0.3	0.17	0.39	0.2	0.19	1.05	0.28	-162.92	157.06
run17	2.72	0.61	0.8	0.39	0.94	0.8	0.54	1.09	0.18	0.32	0.18	0.42	0.21	0.18	1.05	0.27	-166.71	151.9
run18	2.74	0.61	0.79	0.4	0.89	0.8	0.49	1.24	0.18	0.32	0.18	0.43	0.2	0.19	1.07	0.27	-148.49	147.3
run19	2.73	0.62	0.82	0.4	0.93	0.77	0.54	1.09	0.18	0.33	0.18	0.42	0.21	0.2	1.03	0.27	-131.54	149.69
run20	2.74	0.63	0.78	0.4	0.95	0.76	0.53	1.08	0.18	0.3	0.18	0.42	0.21	0.19	1.04	0.28	-150.95	150.79
run21	2.72	0.64	0.82	0.39	0.93	0.8	0.52	1.09	0.19	0.31	0.17	0.41	0.2	0.19	1.05	0.28	-163.88	151.64
run22	2.73	0.61	0.78	0.39	0.92	0.81	0.53	1.08	0.18	0.32	0.17	0.42	0.21	0.18	1.07	0.28	-164.43	144.9
run23	2.73	0.62	0.79	0.39	0.83	0.76	0.52	1.13	0.19	0.31	0.18	0.43	0.21	0.18	1.05	0.28	-164.11	151.2
run24	2.74	0.74	0.88	0.4	0.92	0.81	0.49	1.2	0.35	0.23	0.18	0.29	0.21	0.18	1.07	0.28	-163.43	149.93
run25	2.72	0.63	0.79	0.38	0.88	0.74	0.56	1.1	0.19	0.34	0.18	0.41	0.2	0.19	1.04	0.28	-116.97	146.64
run26	2.72	0.61	0.79	0.4	0.89	0.83	0.52	1.12	0.19	0.3	0.18	0.42	0.21	0.18	1.06	0.28	-166.16	140.06
run27	2.71	0.69	0.78	0.4	0.82	0.8	0.53	1.24	0.19	0.32	0.18	0.43	0.21	0.18	1.05	0.28	-162.65	150.29
run28	2.74	0.61	0.77	0.4	0.92	0.78	0.53	1.23	0.18	0.32	0.18	0.43	0.21	0.18	1.05	0.28	-148.24	150.09
run29	2.71	0.73	0.88	0.38	0.92	0.84	0.53	1.03	0.32	0.24	0.17	0.31	0.2	0.18	1.05	0.28	-131.87	146.6
run30	2.74	0.65	0.81	0.4	0.88	0.78	0.56	1.24	0.19	0.3	0.18	0.43	0.21	0.18	1.04	0.28	-149.43	150.91
run31	2.71	0.64	0.77	0.4	0.96	0.82	0.53	1.25	0.18	0.31	0.17	0.42	0.21	0.18	0.99	0.28	-158.86	150.5
run32	2.72	0.6	0.78	0.38	0.89	0.82	0.53	1.19	0.18	0.31	0.18	0.43	0.2	0.19	0.99	0.28	-149.01	152.9
run33	2.73	0.59	0.79	0.4	0.91	0.76	0.52	1.11	0.18	0.32	0.17	0.42	0.21	0.19	1.0	0.27	-148.76	149.11
run34	2.77	0.64	0.78	0.4	0.89	0.83	0.54	1.24	0.19	0.33	0.19	0.43	0.21	0.18	1.03	0.27	-149.98	145.62
run35	2.73	0.62	0.81	0.39	0.93	0.79	0.55	1.07	0.18	0.33	0.17	0.42	0.21	0.19	1.03	0.28	-164.43	141.47
run36	2.75	0.64	0.82	0.39	0.95	0.76	0.52	1.08	0.18	0.32	0.17	0.41	0.2	0.19	1.06	0.27	-165.8	148.06
run37	2.73	0.64	0.83	0.38	0.96	0.76	0.55	1.11	0.22	0.3	0.17	0.38	0.21	0.2	1.07	0.28	-144.23	130.64
run38	2.71	0.62	0.77	0.39	0.99	0.78	0.51	1.25	0.18	0.32	0.17	0.42	0.21	0.19	1.03	0.27	-145.39	151.66
run39	2.67	0.62	0.81	0.38	0.92	0.75	0.55	1.09	0.18	0.32	0.17	0.41	0.2	0.19	1.04	0.27	-164.67	148.58
run40	2.75	0.71	0.87	0.39	0.9	0.79	0.53	1.16	0.33	0.24	0.17	0.3	0.21	0.18	1.07	0.28	36.3	61.5
run41	2.76	0.65	0.8	0.38	0.89	0.81	0.53	1.06	0.18	0.32	0.18	0.43	0.22	0.19	1.04	0.27	-119.08	143.61
run42	2.74	0.67	0.82	0.38	0.81	0.76	0.53	1.13	0.22	0.27	0.17	0.41	0.21	0.19	1.03	0.28	-98.64	147.45
run43	2.74	0.6	0.79	0.39	0.95	0.8	0.53	1.18	0.18	0.32	0.17	0.42	0.21	0.19	1.05	0.27	-146.06	146.37
run44	2.74	0.62	0.79	0.4	0.91	0.84	0.49	1.24	0.18	0.29	0.18	0.43	0.2	0.18	1.02	0.27	-131.91	145.61
run45	2.67	0.62	0.79	0.39	0.91	0.84	0.53	1.19	0.18	0.31	0.17	0.42	0.2	0.18	0.99	0.28	-162.12	149.31
run46	2.73	0.61	0.78	0.38	0.94	0.79	0.53	1.08	0.18	0.32	0.17	0.42	0.21	0.18	1.05	0.27	-31.91	144.47
run47	2.73	0.62	0.81	0.4	0.96	0.7	0.56	1.18	0.19	0.33	0.17	0.41	0.19	0.19	1.04	0.29	-165.88	147.11
run48	2.75	0.63	0.81	0.39	0.96	0.77	0.51	1.21	0.2	0.31	0.17	0.4	0.2	0.18	1.01	0.28	-165.3	150.54
run49	2.73	0.65	0.83	0.39	0.93	0.8	0.54	1.12	0.18	0.33	0.18	0.42	0.22	0.2	1.06	0.27	-147.62	151.2
run50	2.73	0.64	0.81	0.4	0.88	0.83	0.51	1.23	0.19	0.3	0.2	0.45	0.23	0.18	1.08	0.28	-114.69	150.83

Supplementary Figure S1. Example heat table from the closing process, visualizing the progression of one round of AS simulations towards the target structure.

Rows correspond to 50 individual simulations of one round of AS. Top row: list of structural features including distances, angles, and ϕ/ψ angles. Second row: reference (target) values of the features, here taken from the 6I3P structure of Prp43•U_7•ATP. Third row: starting values of the features, taken from the open simulation frame. Columns show the feature values at the end of this AS round. The color indicates the similarity with the starting feature (purple) or with the reference/target feature (yellow). Such tables have been used extensively to monitor the progression of the AS simulations and to select the most successful simulation to be used a seed for the next round of AS.

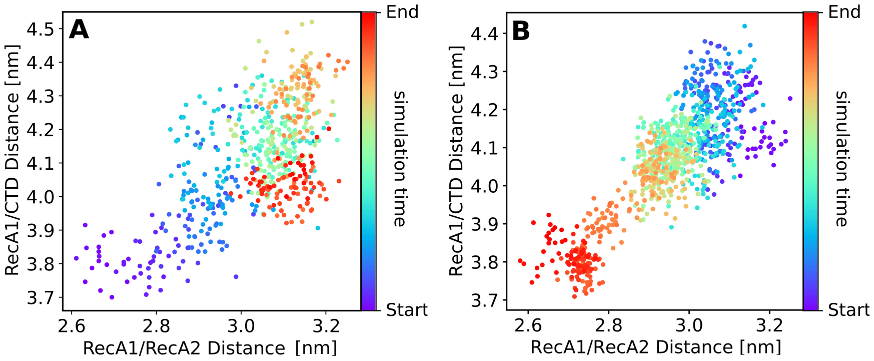

Supplementary Figure S2. Center-of-mass distances between C-terminal domain relative to RecA-like domains.

(A) RecA1–CTD distance versus RecA1–RecA2 distance during opening and (B) during closing. Here, the center of mass of CTD was defined with the helices S544–S555, D583–R600, and Y615–K629. The color indicates the cumulative simulation time from start (purple) to end (red). During both the opening (A) and (B) closing process, the RecA1–CTD distance was correlated with the RecA1–RecA2 distance, illustrating that the CTD domain moved concertedly with RecA2 along the RNA, as shown in Figure 2 in the paper.

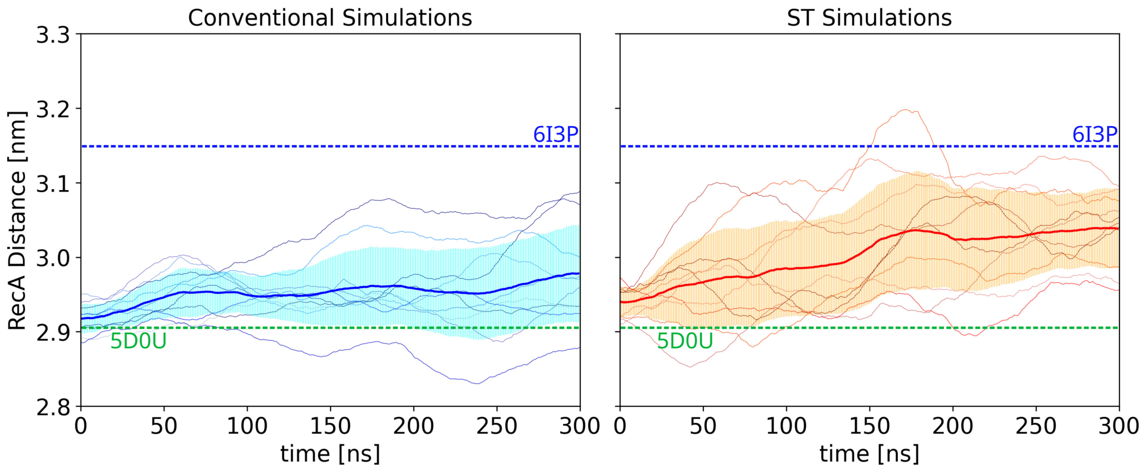

Supplementary Figure S3. Accelerated conformational sampling with simulated tempering (ST).

RecA1–RecA2 distance of Prp43 after removal of ADP in conventional simulations (left) or ST simulations (right). Among 10 conventional simulations, only two simulation reached a semi-open state within 300 ns, as indicated by the RecA1/RecA2 distance of over 3.0 nm. In contrast, among 10 ST simulations, four reached the semi-open state within only 100 ns and six simulation reached the semi-open state within 300 ns. In addition, four ST reached a fully open state indicated by a distance larger 3.1 nm. The more rapid increase of the average (thick lines) and of standard deviations (shaded areas) in ST compared to conventional simulations indicate accelerated sampling of the conformational space within a short simulation time.

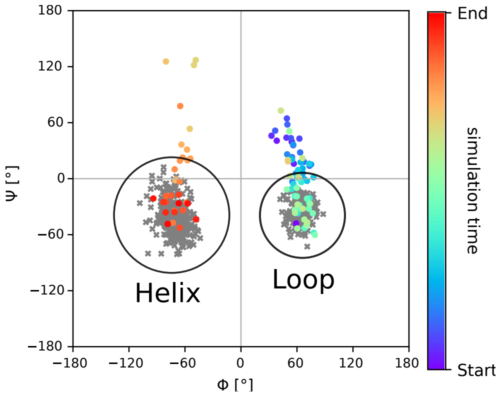

Supplementary Figure S4. Ramachandran plot of sensor serine S387.

Grey crosses: ϕ and ψ angles of S387 during free 1 μs simulations starting from the 6I3P structure (left), corresponding to the open conformation, or starting from the 5LTA structure (right), corresponding to the closed conformation. According to the ϕ/ψ angles, S387 remained in the helical or in a loop state in the simulations of the open or closed state, respectively. No transition was observed. Colored dots: ϕ/ψ angles during a AS simulation with a successful loop-to-helix transition of the sensor serine. The color indicates the simulation time of the successful AS run from purple to red.

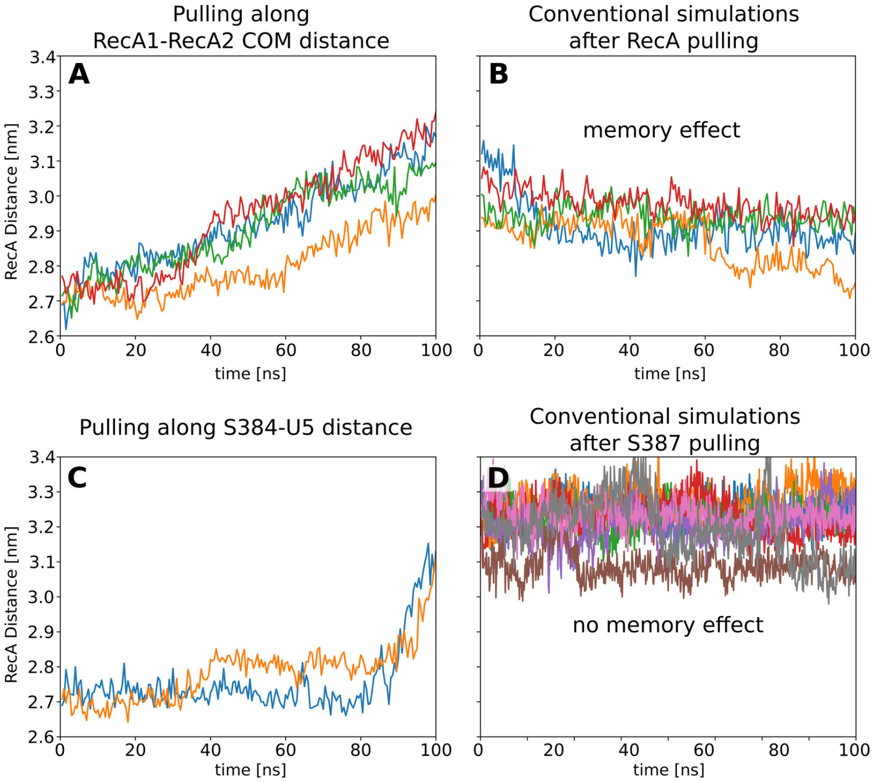

Supplementary Figure S5. On the correlation between Prp43 opening and loop-to-helix transition of the S387–G392 sensor loop.

RecA1–RecA2 distance versus simulation time during (A/C) pulling simulations and (B/D) after releasing the pulling restraint. (A) Pulling along the RecA1–RecA2 center-of-mass (COM) distance leads to major memory effects, as shown by (B) the partial re-closure of the RecA1/RecA2 interface after release of the restraint. (C) Pulling along the S387–U5 distance, thereby driving the loop-to-helix transition of the sensor loop, leads to opening of the RecA1–RecA2 interface and (D) does not lead to memory effects. The absence of memory effects after the loop-to-helix transition suggests that the sensor loop transitions are critical for Prp43 opening and closing.

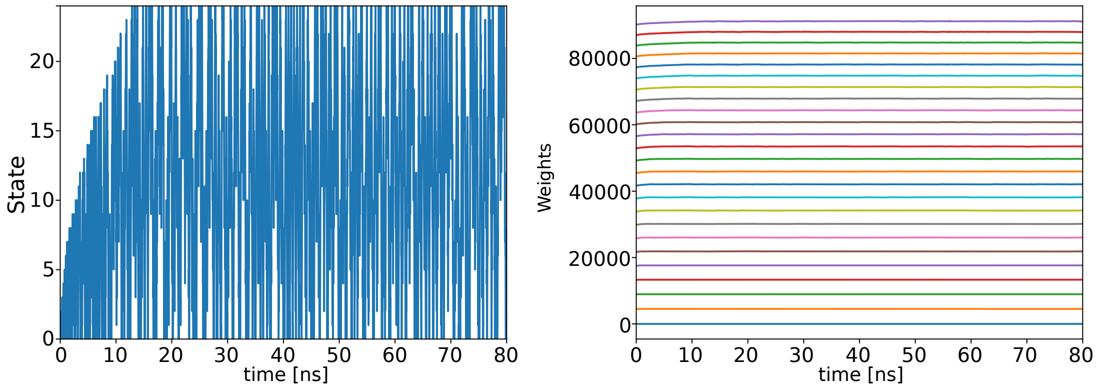

Supplementary Figure S6. Coverage of temperature states during simulated tempering. Left: temperature state versus simulation time during an example simulated tempering (ST) simulation. During the burn-in phase within the first 17 ns, states with increasingly higher temperature were visited, reflecting the gradual adaptation of the weights. Right: weights of temperature states versus simulation time. Weights were only slightly adapted at the beginning of the simulation, reflecting good initial weights. After 17 ns, the weights were converged, and all states were frequently visited.

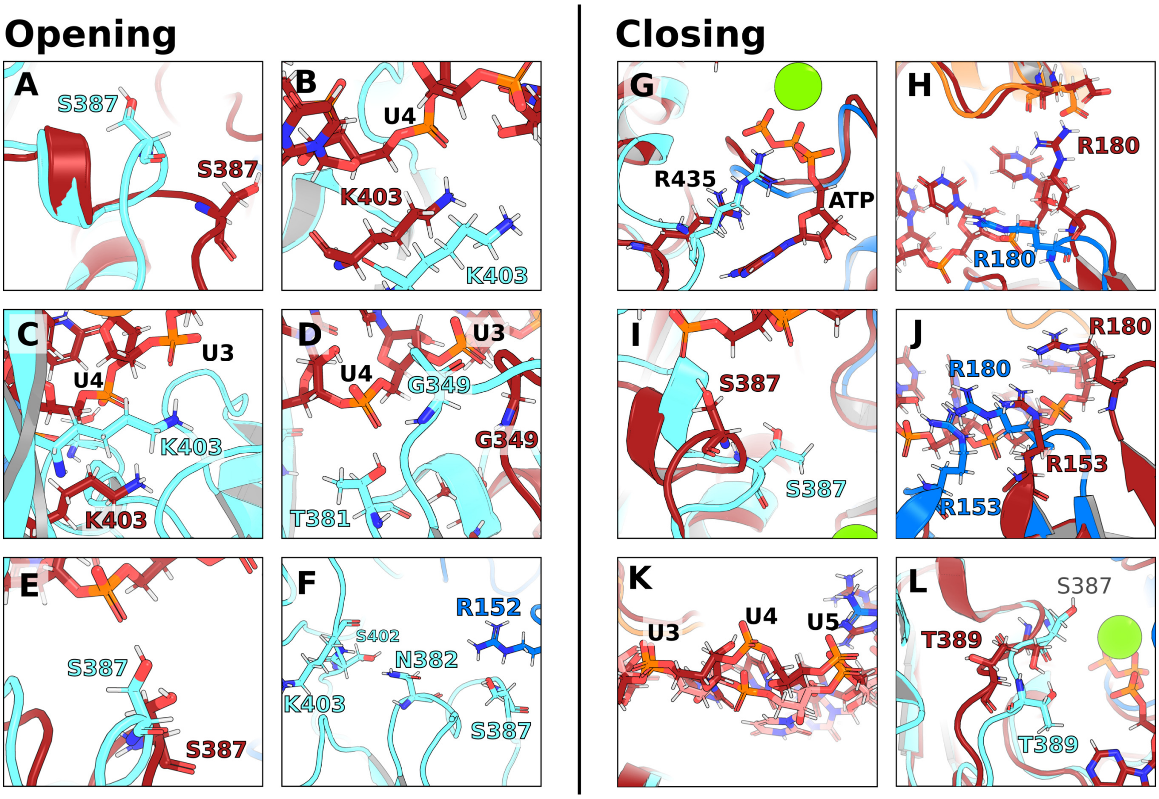

Supplementary Figure S7. Transitions of molecular switches contributing to the mean first passage times.

Opening: (A) 1st transition: S387 flip from loop (red) to helical state (cyan). (B) 2nd transition: K403 connected to U4 (red) to disconnected to U4 (cyan). (C) 3rd transition: K403 disconnected to U3 (red) to connected to U3 (cyan). (D) 4th transition: Hook-loop shift from U3 (G349; red) to U4 (G349 and T381; cyan). (E) 5th transition: S387 not connected to U5 (red) to S387 connected to U5 (cyan). (F) 6th transition: H-bond network after successful opening. RecA2 is connected with RecA1 with an H-bond between R152 and S387. The N382 of the sensor loop is connected with the β-hairpin via K403 and S402.

Closing: (G) 1st transition: R435 binding to β and γ phosphates of ATP (from red to cyan). (H) 2nd transition: R180 bound to CTD (red) to disconnected from CTD (blue). (I) 3rd transition: S387 flip from helical (red) to loop (cyan) state. (J) 4th transition: Hook-turn shift from U6/U7 (red) to U5/U6 (blue). (K) 5th transition: RNA rotation at U4 (from pink to red). (L) 6th transition: Bending of S387 and T389 during the end of the closing process (from red to cyan), finalizing the formation of the RecA1/RecA2 interface corresponding to a RecA distance of 2.65 nm.

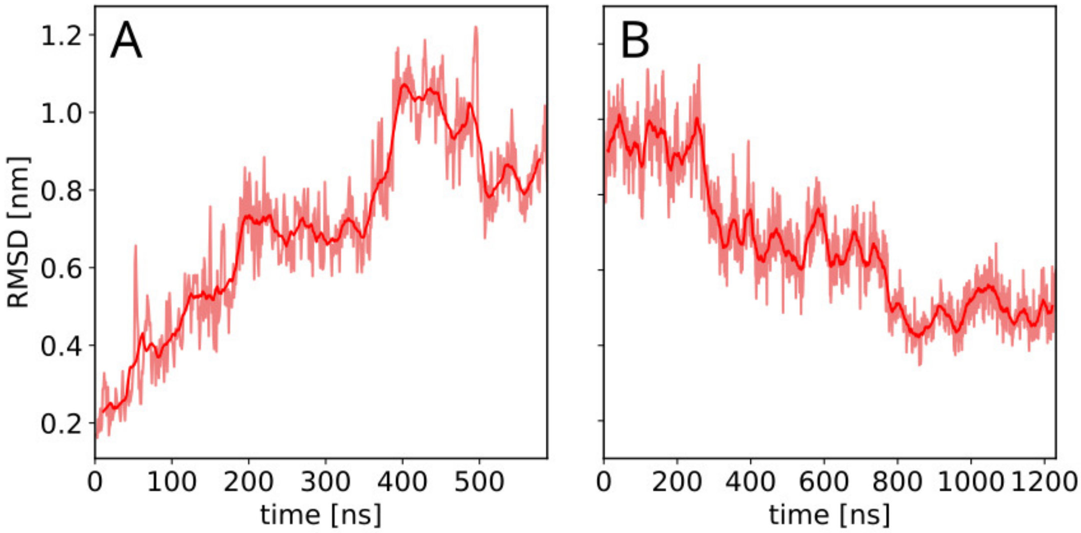

Supplementary Figure S8. Backbone RMSD relative to the closed Prp43 conformation during opening and closing, after superimposing the backbone atoms of the RecA1 domain. (A) RMSD of the opening trajectory and (B) of the closing trajectory relative to the starting frame of the opening process.

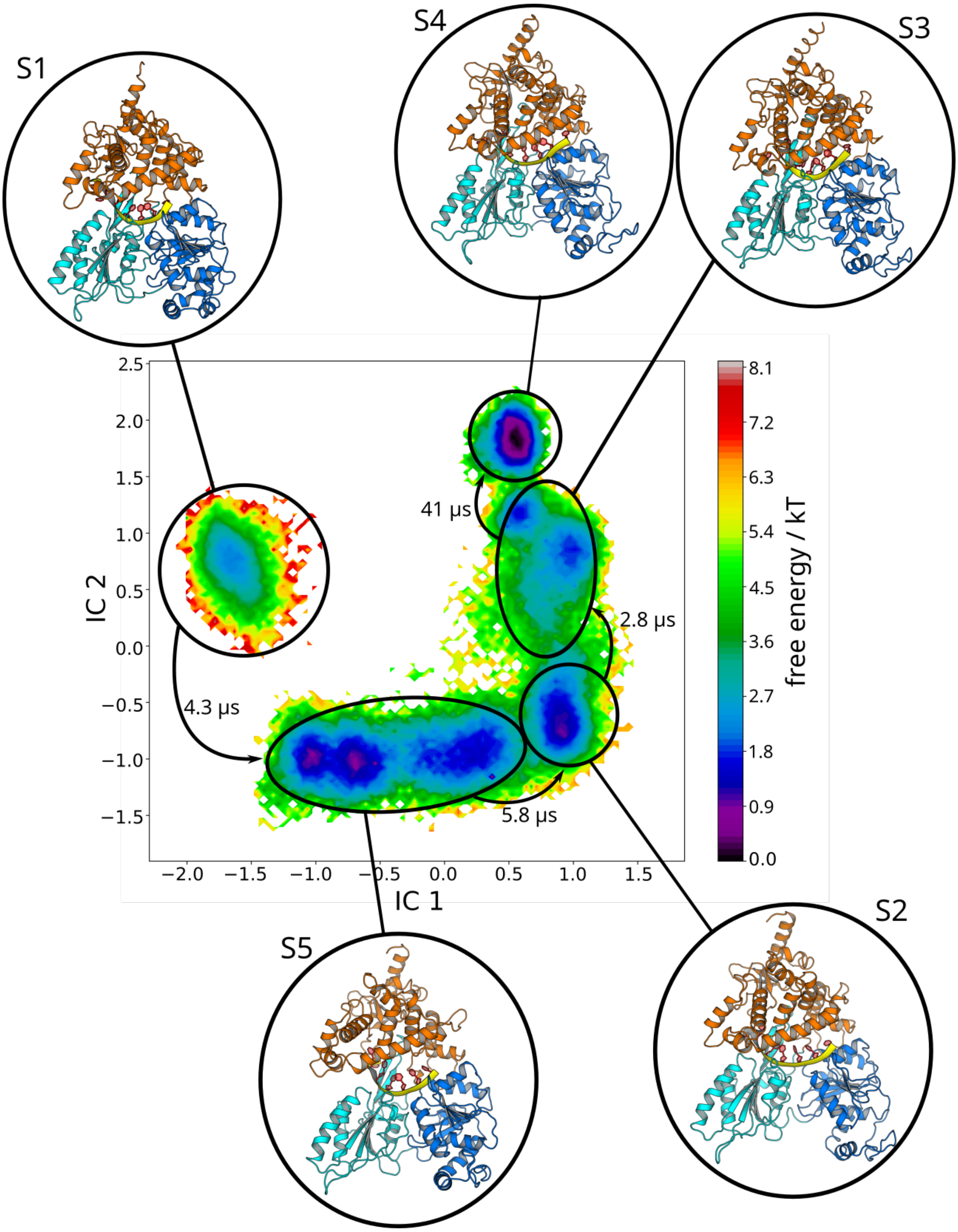

Supplementary Figure S9. Estimate of the free energy landscape calculated from the stationary distribution during the construction of the MSM. Five macrostates are encircled with black lines, each connected with a representative Prp43 conformation. The translocation path is indicated by arrows with the corresponding MFPT from state to state as suggested by the MSM. The metastable states corresponding to the free energy basins are clearly revealed by the MSM, however, the MFPT between states S1 and S5 is likely underestimated, as revealed by the comparison with the liner kinetic model (see main Text).

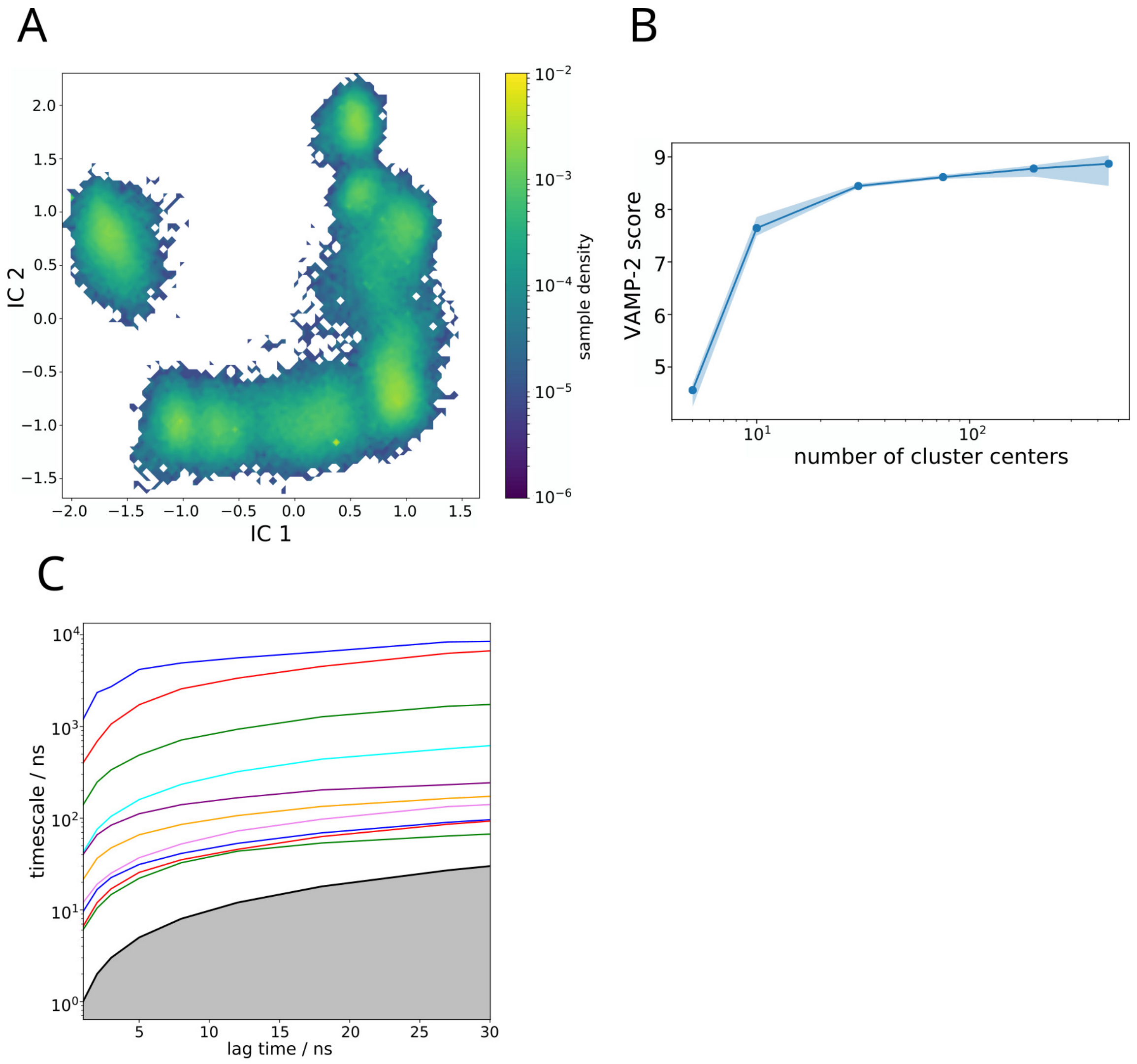

Supplementary Figure S10. Further analysis and validation of MSM. (A) tiCA plot of the first two independent components (ICs). (B) VAMP2 score versus the number of cluster centers for the microstate assignment. The VAMP2 score converges for more than 80 cluster centers. (C) Implied timescales versus lag time. The time scales converge for lag times greater than approximately 20 ns.

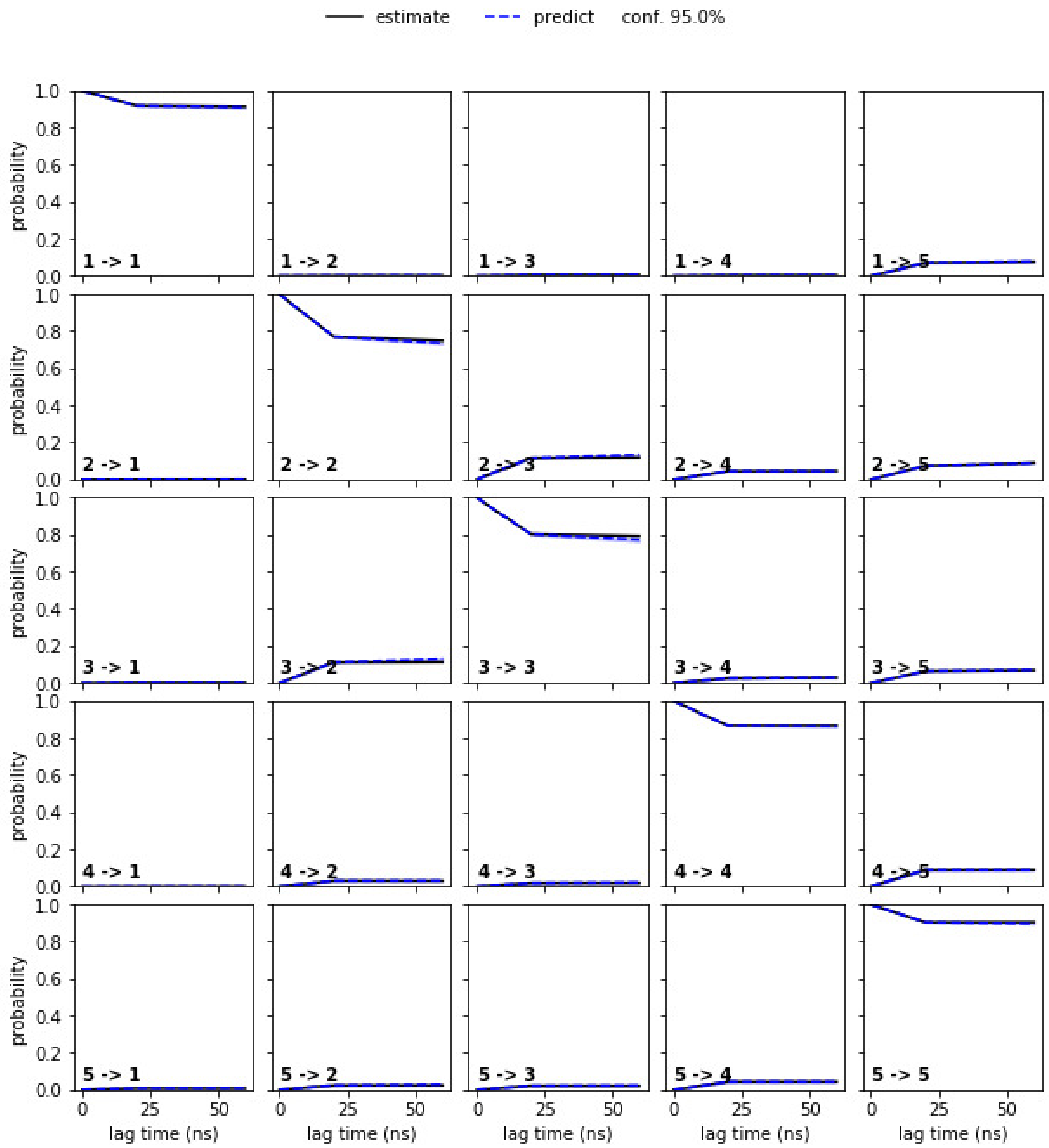

Supplementary Figure S11. Chapman-Kolmogorov test for the validation of macrostates of the MSM.

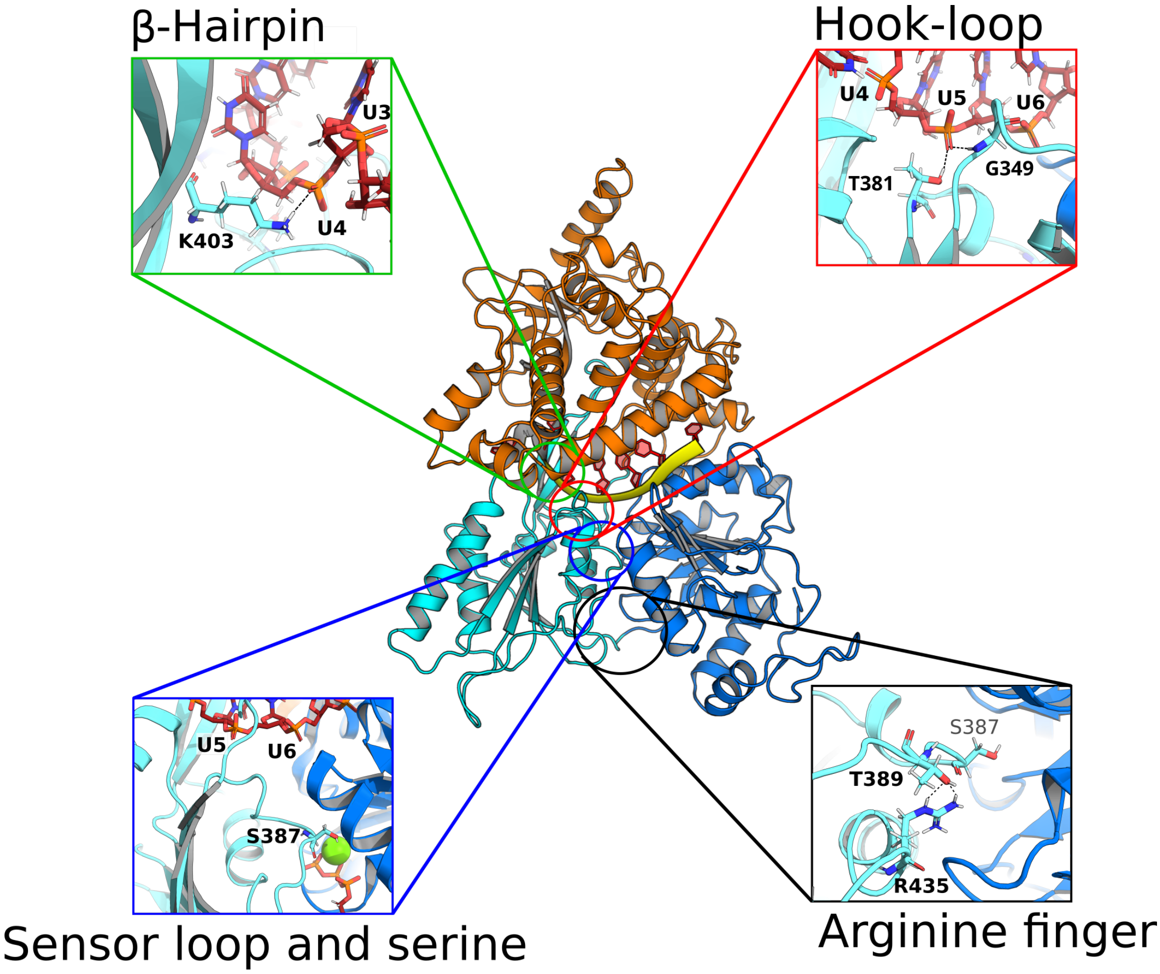

Supplementary Figure S12. Close-up views of structural features analyzed in Figure 3 (opening process), highlighting the feature locations within the overall Prp43 structure protein structure.

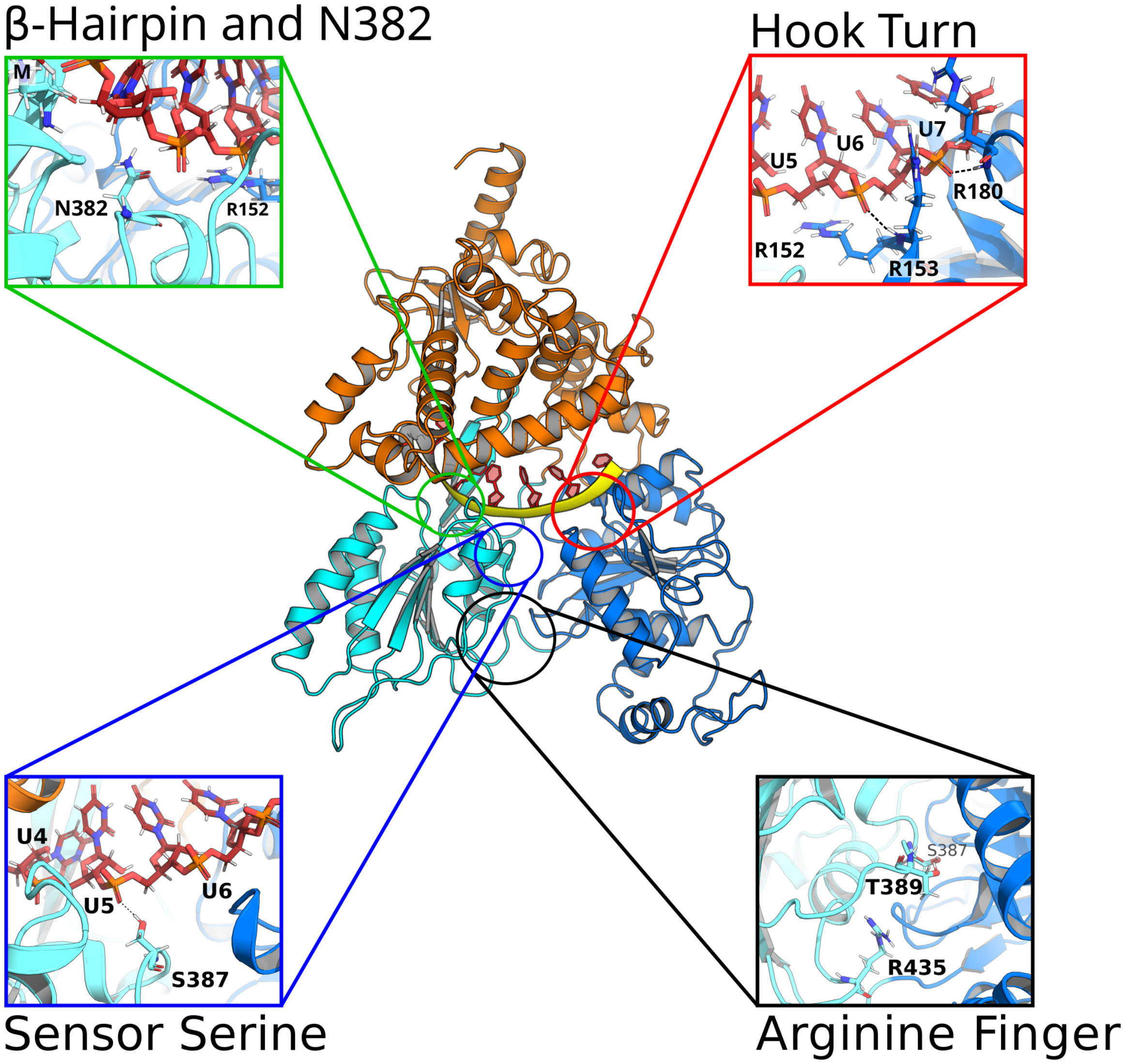

Supplementary Figure S13. Close-up views of structural features analyzed in Figure 5 (closing process), highlighting the feature locations within the overall Prp43 structure protein structure.

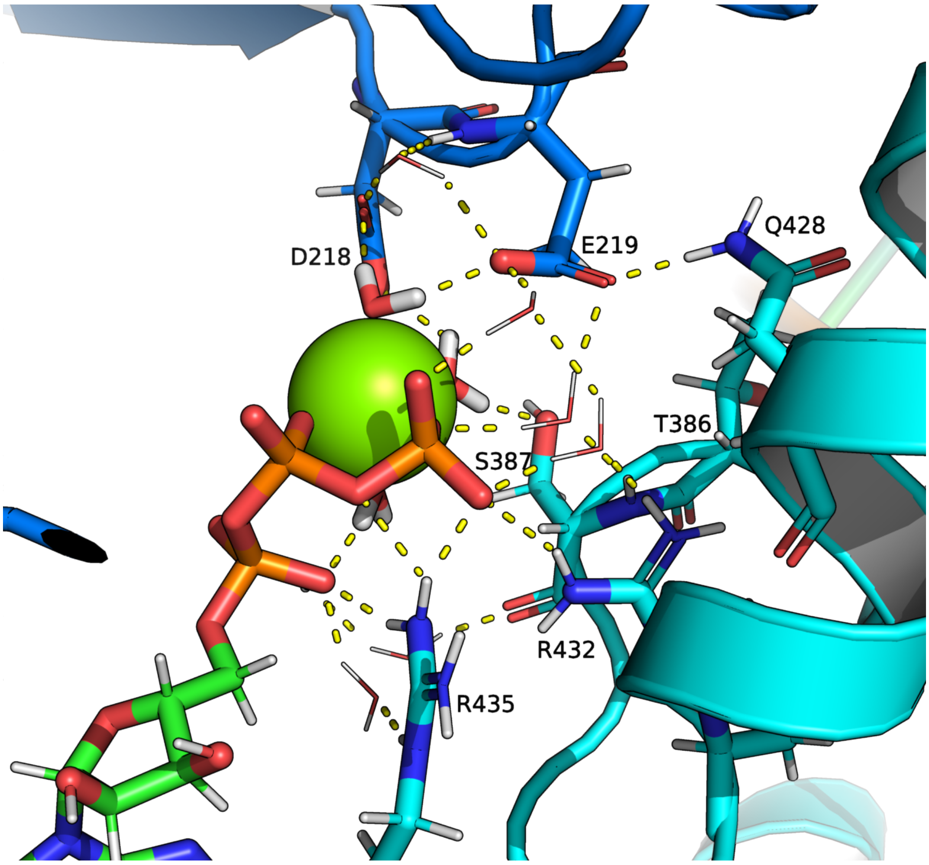

Supplementary Figure S14. Close-up view on the water complex and network during the end of the closing process. The network is similar to the network proposed by Tauchert *et al.* for the closed crystal structure 5LTJ. However, the water molecule in position for a S_N2 attack shown by Tauchert *et al.* was not observed in our simulations. Three water molecules coordinating the Mg^{2+} ions are shown as thick sticks, all other water molecules as thin sticks.

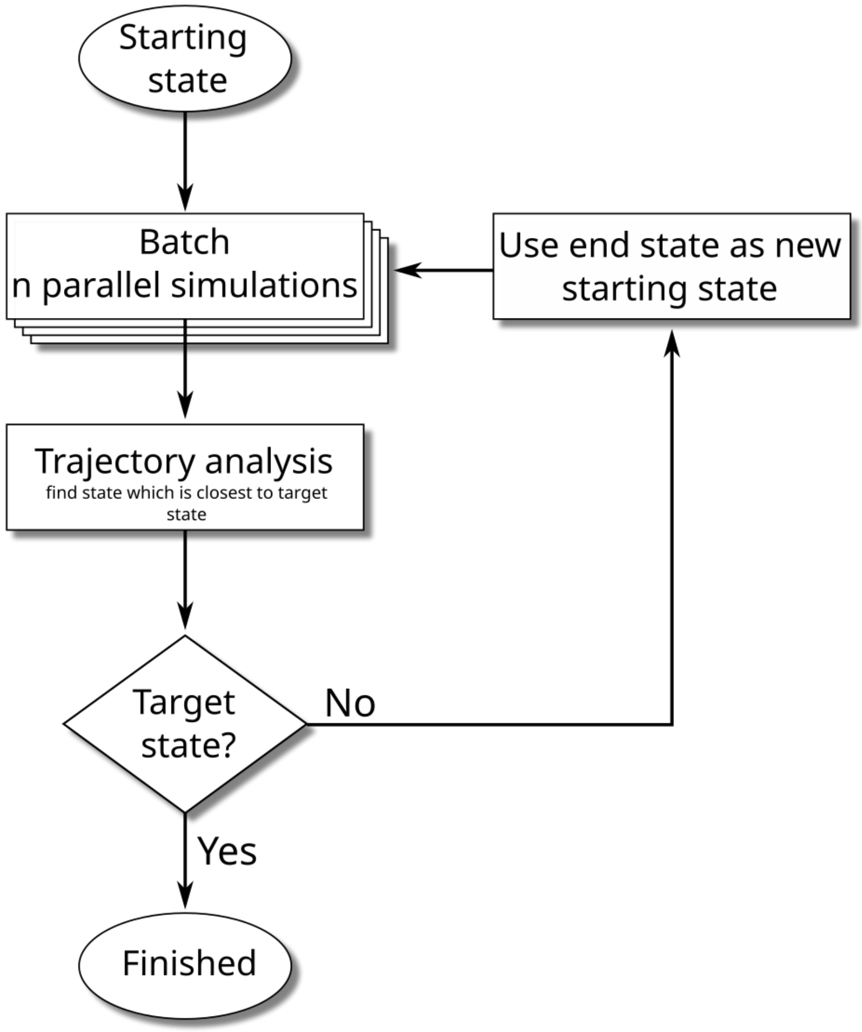

Supplementary Figure S15. Flowchart for our adaptive sampling protocol. 1. Run n parallel simulations from the same starting state. 2. Analysis of the trajectory in terms of pre-defined features (Fig. S1) as well as visual inspection. 3. Identify one or few most successful simulations. Here, "most successful" refers to the furthest progression along the pre-defined features. 4. Have all features reached their target values with reasonable accuracy? If not, run another batch of parallel simulations starting from the most successful simulation(s). If yes, the overall transition (Prp43 opening or closing) is considered as successful.

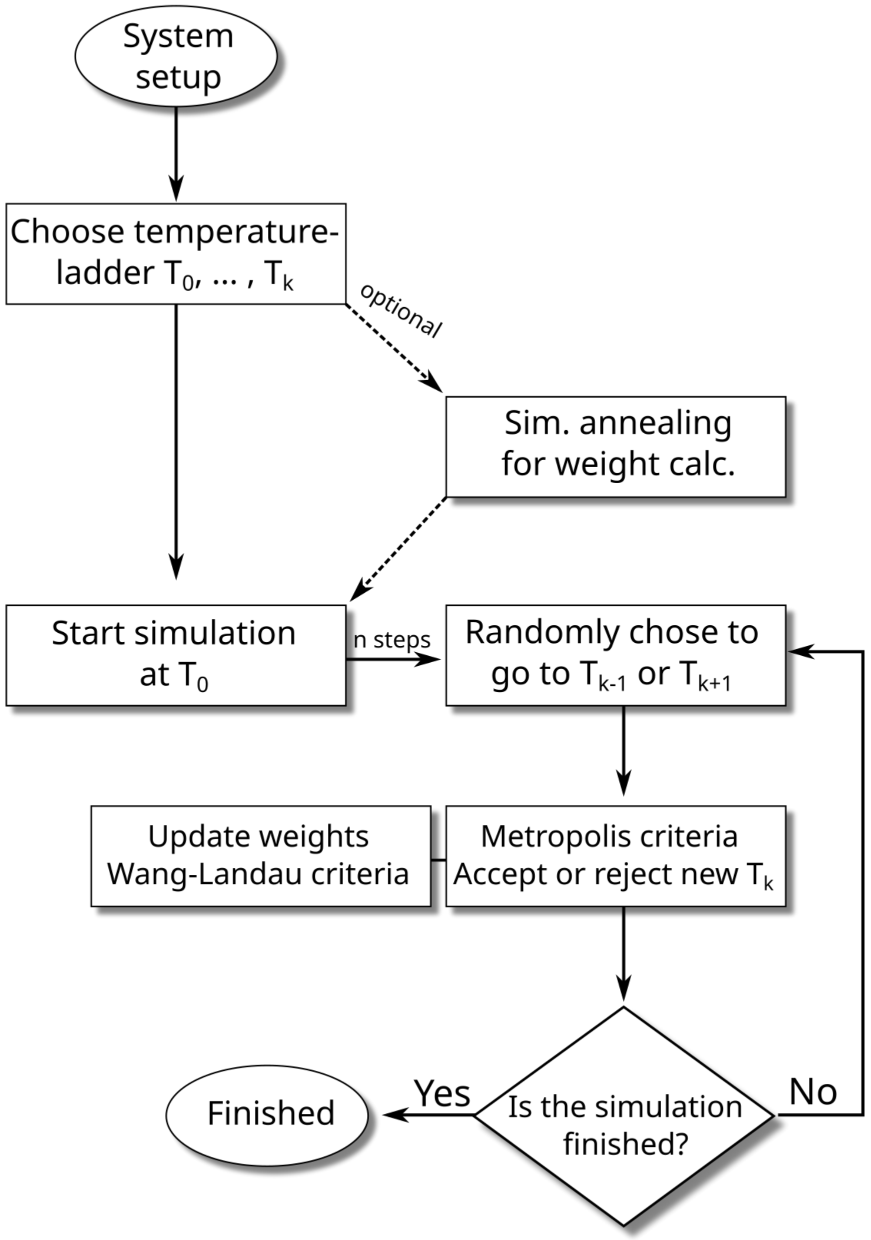

Supplementary Figure S16. Simplified flow chart for the simulated tempering method. 1. Specify temperature ladder $T_0, ..., T_k$. In our specific case, we used $T_n = (300, ..., 348)$K (1.5 Optional: Initial weights for each temperature state may be estimated following Pande *et al.* using a simulated annealing simulation. [1]) 2. Start the simulation from the ground temperature T_0. 3. Randomly propose a temperature move to T_{k-1} or T_{k+1} after a pre-selected number of steps. 4. Accept or reject the move with a Metropolis criterion. Simultaneously, update the weights of temperature states with the Wang-Landau method to maintain a uniform occupancy over temperature states. 5. Check if the simulation has reached the maximum number of steps. If not, propose the next temperature move and proceed. If yes, the simulation is finished.

[1] Park, S.; Pande, V. S. Choosing weights for simulated tempering. *Phys. Rev. E* **2007**, *76*, 016703.
