# Supplementary material for: Continuous millisecond conformational cycle of a DEAH box helicase reveals control of domain motions by atomic-scale transitions
Source: Commun Biol. 2023 Apr 7;6:379. doi: 10.1038/s42003-023-04751-z (PMC10082070; doi:10.1038/s42003-023-04751-z)
Supplement: Supplementary file 3 — Description of Additional Supplementary Files [file 42003_2023_4751_MOESM3_ESM.pdf]

Description of Additional Supplementary Files

File name: Supplementary Movie 1

Description: Movie of the dynamics of RNA and the molecular switches during the complete process.

File name: Supplementary Movie 2

Description: Movie of the concatenated opening and closing process of the complete enzyme.

File name: Supplementary Movie 3

Description: Movie of the projected first eigenvector of the PCA of the isolated RecA1 domain.

File name: Supplementary Movie 4

Description: Movie of the projected first eigenvector of the PCA of the isolated RecA2 domain.
